# O087: An acinetobacter spp. (GIM-1) pseudo-outbreak due to contamination of a pneumatic transport system (PTS) in a large university hospital

**DOI:** 10.1186/2047-2994-2-S1-O87

**Published:** 2013-06-20

**Authors:** BC Gärtner, S Jungmann, A Dawson, A Halfmann, C Petit, M Kaase, SG Gatermann, M Klotz, L von Müller, P Lüttchens, R Veith, M Herrmann

**Affiliations:** 1 Institute of Medical Microbiology and Hygiene, University of the Saarland, Homburg/Saar, Germany; 2 National Reference Laboratory for Gram-negative Bacilli, University of Bochum, Bochum, Germany; 3 Technical Department, University of the Saarland, Homburg/Saar, Germany

## Introduction

In clinical specimen from 21 patients, two species of *Acinetobacter baumannii complex* were isolated with resistance to betalactams including carbapenems. None of the patients had symptoms indicative for infection. Patients were treated in various departments and a large spectrum of age, clinical specimen, and underlying disease was observed.

## Objectives

To identify the source of transmission.

## Methods

Molecular analysis of the isolated strains and extensive epidemiologic workup.

## Results

Molecular analysis confirmed presence of a metallobetalactamase of GIM-1 genotype. Extended epidemiologic workup, however, did not reveal any factors for transmission, previous hospitalizations, common places of residence moreover Extensive analysis of specimen workup in the laboratory rendered a laboratory associated pseudo-outbreak highly unlikely.

Identical *Acinetobacter* spp (GIM-1) was found on 8/13 patient data sheets accompanying the positive specimen, as well as PTS cartridges prospectively analyzed. Positive microbiological results were associated with humidity in the cartridges. Moreover the outbreak strain was detected in air samples close to the PTS.

Upon identification of the PTS as cause of the pseudo-outbreak, all clinical infection control measures were lifted, yet, rigorous measures for hygienic handling of PTS stations, cartridges, and transported specimens were introduced. Moreover, the PTS was decontaminated with disinfectants.

## Conclusion

To our knowledge, this is the worldwide first description of a large-scale contamination of a hospital PTS. In absence of established environmental hygiene standards, bacterial contamination of PTS may occur for extended time periods prior to recognition, and may be the cause not only for pseudo-outbreaks but also for transmission of nosocomial pathogens with yet unknown consequences. This experience suggests that establishment of standards is necessary for the particular challenge which PTS may represent for hospital hygiene.

## Disclosure of interest

None declared.

